# Absence of short-term changes in knowledge and attitudes among household contacts of COVID-19 cases during the post-acute phase of the pandemic in Catalonia and Navarre, Spain

**DOI:** 10.3389/fpubh.2024.1306284

**Published:** 2024-02-29

**Authors:** Vanessa Bullón-Vela, Diana Toledo, Aitziber Echeverría, Pere Godoy, Manuel García Cenoz, Ignasi Parrón, Jesús Castilla, Angela Domínguez, Iván Martínez-Baz

**Affiliations:** ^1^Instituto de Salud Pública de Navarra, Pamplona, Spain; ^2^Instituto de Investigación Sanitaria de Navarra (IdiSNA), Pamplona, Spain; ^3^CIBER Epidemiología y Salud Pública (CIBERESP), Madrid, Spain; ^4^Facultat de Medicina, Universitat de Barcelona, Barcelona, Spain; ^5^Facultat de Medicina, Universitat de Lleida, Catalonia, Spain; ^6^Institut de Recerca Biomèdica de Lleida (IRBLleida), Catalonia, Spain; ^7^Departament de Salut, Generalitat de Catalunya, Barcelona, Spain

**Keywords:** knowledge, attitude, COVID-19, household contacts, preventive measures

## Abstract

**Objectives:**

To evaluate short-term changes in knowledge and attitude towards COVID-19 and preventive measures during the post-acute phase of the pandemic in Spain.

**Methods:**

A survey was performed in Catalonia and Navarre between May-2022 and July-2023 in household contacts of COVID-19 cases. Knowledge and attitude were assessed at baseline and at three months, using a Likert scale. Responses were grouped according to correct or incorrect.

**Results:**

At baseline, 172 subjects were contacted, 118 (69%) of which completed a follow-up interview three months later. Knowledge of correct hand-washing and mask protocols had maintained over time (−1.7%, *p* = 0.553 and − 2.5%, *p* = 0.473, respectively). Attitudes toward preventive measures was adequate in the first interview (86%), but attitudes regarding use of face masks decreased significantly (−9.1%, *p* = 0.048) over time in participants with higher risk of severe COVID-19. However, most short-term changes in knowledge and attitudes were not statistically significant.

**Conclusion:**

Household contacts showed correct knowledge and attitude towards COVID-19 and its preventive measures, without significant changes in the short term despite a relaxation of government-mandated preventive measures. These results provide relevant information in case of a new health emergency due to respiratory viruses.

## Introduction

The coronavirus disease 2019 (COVID-19) is an infectious disease that has quickly spread across the world (1), and in 2020 was declared a global pandemic disease by the World Health Organization (WHO) (2), resulting in the worldwide implementation of government-regulated strategies for its containment. During 2022, many countries adopted strategies for the de-escalation of preventive measures, and in 2023 the transition beyond the acute phase of the pandemic began (3). In this context, the European Centre for Disease Prevention and Control (ECDC) strongly recommends that governments monitor pandemic trends, focusing on the prevention and management of SARS-CoV-2 variants and the long-term health consequences caused by COVID-19 (4).

Several policy efforts to reduce the negative effects of COVID-19 have focused on reducing the virus’ circulation using a range of methods to prevent its transmission and improve diagnosis. These range from non-pharmaceutical interventions through to extensive vaccination programs (2, 5, 6). These containment measures are implemented by Governments in different ways, depending on various factors including SARS-CoV-2 variant dynamics (transmissibility and immune escape properties) and epidemiological situation (6, 7).

Human COVID-19 transmission occurs through contact with infected respiratory droplets either through direct contact with the eyes, nose or mouth, or indirectly after contact with an infected surface. In both cases the probability of transmission is higher in people who meet the criteria of close contact. However, household contacts are even more at risk (as a shared dwelling is a highly favorable setting for COVID-19 transmission), due to their exposure to the disease being usually more intense and repeated (8, 9).

Subjects’ knowledge of correct procedures to prevent transmission in this context is clearly of key importance to prevent infection. Better knowledge of the COVID-19 disease and its prevention protocols are associated with well-informed decisions and adequate adherence to preventive health measures to counter COVID-19 (10). Additionally, although the success of any containment measures and protocols depends on the willingness of individuals to follow those measures, this has also been strongly linked to adequate knowledge and attitudes towards the disease (11). There is still not sufficient understanding attitudes, knowledge sources, and other drivers that could promote and reinforce appropriate preventive measures, nor how and why these drivers might change over time in the post-acute pandemic phase (3).

This study aims to evaluate short-term changes in knowledge and attitudes about the COVID-19 disease and its recommended preventive measures in household contacts of confirmed cases in the post-acute phase of the COVID-19 pandemic in northern Spain, and any changes in the information sources they habitually utilise.

## Methods

### Study design and population

We performed a prospective epidemiological study through a telephone survey. Details of the study design have been described previously (12). In brief, adult household contacts (18 years and over) of confirmed COVID-19 cases were selected in two regions of northern Spain, Catalonia and Navarre, from May 2022 to July 2023. COVID-19 cases were identified and reported by general practitioners from nine primary healthcare centers (one from Navarre and eight from Catalonia) to the Epidemiological Surveillance Services. A random sample of COVID-19 cases were interviewed to identify their household contacts, who were invited to participate in the study. Household contacts with a severe and uncorrectable cognitive or hearing impairment that might impede participants’ ability to complete interviews, and those under 18 years of age were excluded.

Household contacts were defined as permanent residents at the same address of the COVID-19 index case and individuals who had at least two hours of contact with the index case in their place of residence during the transmission period of SARS-CoV-2. Subjects were tested by rapid antigen test after their identification as a contact, and also seven days afterwards by polymerase chain reaction (PCR) in contacts where the first test was negative. Household contacts tested positive for COVID-19 were considered secondary cases (8).

Trained personnel performed a telephone survey on the household contacts in three rounds (baseline, three and six months later). For this study, we only include information from the first two rounds (baseline and three months), to evaluate the possible changes over a short time period.

At baseline, the first part of the telephone interview was to identify the household contact and obtain the patients’ sociodemographic and epidemiological details. In the second part of the interview, subjects’ knowledge and attitudes about the COVID-19 disease and its preventive measures, including vaccination and non-pharmaceutical measures were then collected. The second section was repeated in the subsequent interviews at three- and six-month intervals to measure changes in the variables over time.

### Measurement tool

The process of instrument development and validation has been described elsewhere (12). The questionnaire was developed and adapted from previously validated questionnaires (13, 14) and incorporated the WHO (2), ECDC (5, 6), and Spanish Ministry of Health recommendations (15). A committee of experts in the field develop all sections of the questionnaire to ensure the relevance, consistency, and clarity. The questions regarding to knowledge and attitudes were adapted from other instruments previously published, and a pilot test was performed prior to ensure the viability and reliability of the questionnaire (12). The questionnaire consisted of seven sections and was performed by qualified interviewers.

The first four were related to unchanging data: sociodemographic characteristics (sex, age, educational attainment, and employment status); the presence of major chronic conditions and risk factors (obstructive pulmonary disease, coronary heart disease, diabetes mellitus, and others such as tobacco use, obesity, and hypertension); epidemiological information; and COVID-19 vaccination status.

The final three sections assessed the subjects’ knowledge and attitudes towards COVID-19 disease and its preventive measures, as well as the information sources the subjects commonly used to ascertain information regarding COVID-19. These were assessed using a Likert scale (totally agree, agree, neither agree nor disagree, disagree, and totally disagree).

A total of six items were included in the section evaluating participants’ knowledge of the disease and preventive measures relative to COVID-19 (16, 17). Individuals were asked whether they considered that: asymptomatic and/or vaccinated persons were able to transmit the disease; COVID-19 transmission occurs by means of respiratory droplets from infected individuals; preventive measures such as hand hygiene were effective and; measures such as wearing a face mask, avoiding crowds and avoiding poorly ventilated spaces should be maintained to avoid COVID-19. Correct knowledge was defined if participants answered the categories “agree” or “totally agree” in these questions. For the question referred to all people infected with COVID-19 would become seriously ill; the categories “totally disagree” or “disagree” were considered correct.

The section related to participants’ attitudes included six statements concerning: the compliance of their close friends and family to recommended COVID-19 vaccination and non-pharmaceutical preventive measures, and the effectiveness of the use of facemasks in the community, were considered a positive attitude if subjects answered the categories “agree” or “totally agree.” The preferability or not of getting the disease in order to develop immunity was considered a positive attitude if individuals answer “totally disagree” or “disagree” categories. For the item concerning to high risk of severe illness from COVID-19, it was considered a correct attitude if participants with risk factors (chronic conditions and smoking) answer the categories “agree” or “totally agree” and incorrect understanding if the participant did not have any chronic diseases.

Finally, participants indicated whether they use certain media outlets as sources of information. The outlets included non-official sources such as news channels, written or online press and social media, and official sources including medical professionals, government briefs, and other official sources.

### Statistical analyses

The secondary attack rate (SAR) was measured by baseline characteristics and variables related to the prevention of infection to determine the proportion of COVID-19 cases occurring among susceptible household contacts. Categorical variables were presented as frequencies and percentages. The categories of the Likert scale were grouped into correct/positive or incorrect/negative knowledge or attitude, as previously defined. Absolute changes in knowledge and attitude from baseline to the three-month follow-up were evaluated and the proportion of household contacts who responded correctly at both baseline and in the second round three months later was compared using the chi-square test. Stratified analyses related to knowledge and attitudes were performed on individuals at higher risk of severe COVID-19 considering the presence of medical conditions (major chronic conditions and smoking status) according to the Centers for Disease Control and Prevention (18), or whether the household contacts were infected or not. All statistical tests were 2-sided, and *p*-values of less than 0.05 were considered statistically significant. All statistical analyses were conducted with IBM SPSS version 25.0.

## Results

### Baseline characteristics of household contacts and secondary attack rate

**Figure 1:**
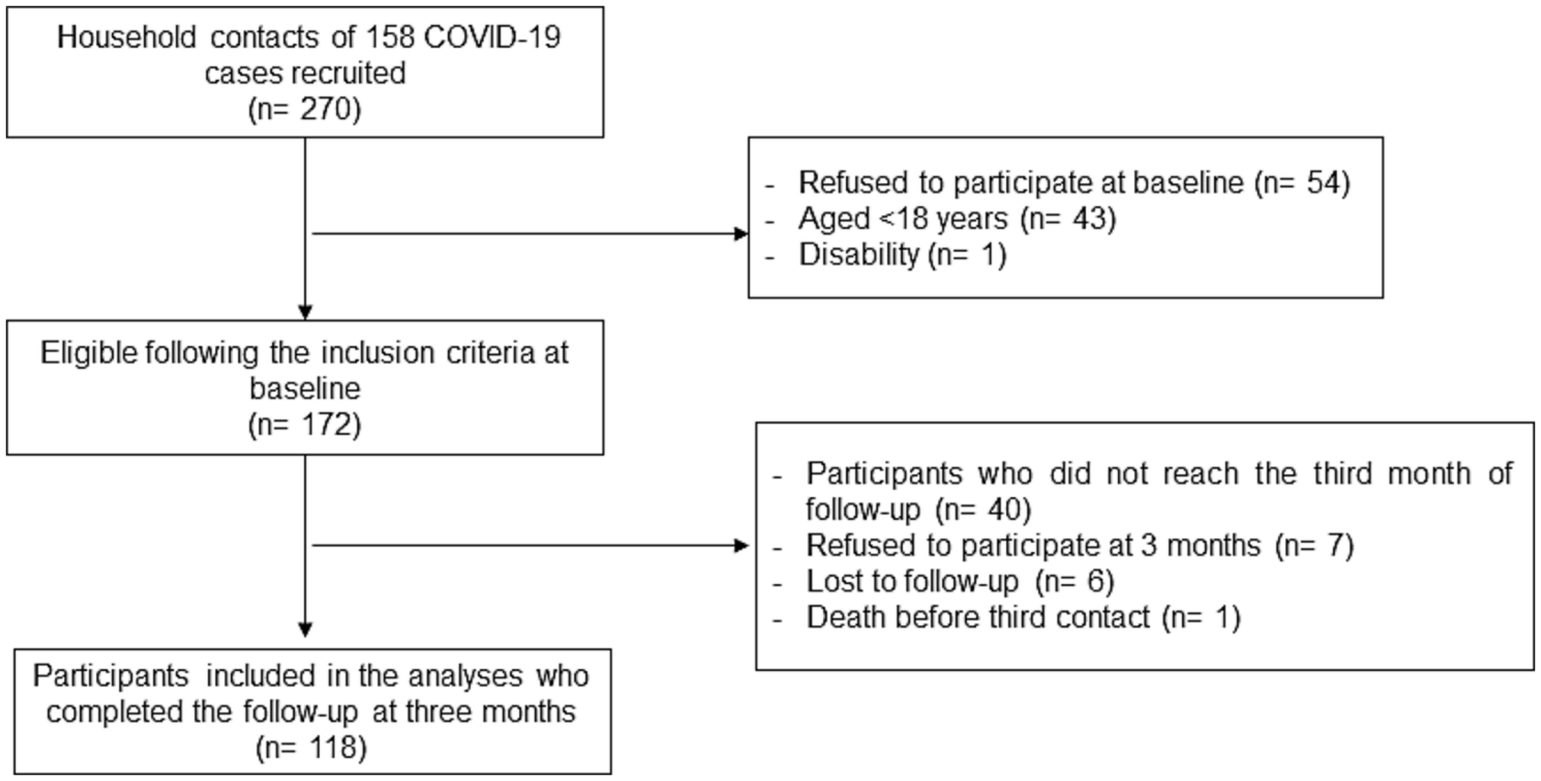
Flow chart summarizing recruitment and retention of study participants (Spain, May 2022 until July 2023).

A total of 270 household contacts were identified during the study period. Finally, after exclusion criteria, 172 household contacts were eligible and completed the baseline questionnaire (Figure 1). Baseline characteristics and secondary attack rates of the participants are presented in Table 1. The majority (71%) of the household contacts were ≤ 64 years old, 58% of them presented risk factors and 33% had some chronic condition.

**Table 1:** Baseline characteristics and secondary attack rates of household contacts of COVID-19 cases (Spain, May 2022 until July 2023).

	Household contacts	Infections	SAR	*p*-value
	*n* (%)	*n*	%	
**Total**	172 (100)	83	48.3	
**Age groups in years**				0.001
18–44	45 (26.2)	18	40.0	
45–64	77 (44.8)	30	39.0	
≥65	50 (29.1)	35	70.0	
**Sex**				0.295
Male	90 (52.3)	40	44.4	
Female	82 (47.7)	43	52.4	
**Education level**				0.153
Primary	28 (16.3)	16	57.1	
Secondary	71 (41.3)	38	53.5	
Higher	72 (41.9)	28	38.9	
Unknown	1 (0.6)	1	100.0	
**Employment status**				0.001
No	78 (45.3)	48	61.5	
Yes	94 (54.7)	35	37.2	
**Risk factors**				0.011
No	73 (42.4)	27	37.0	
Yes	99 (57.6)	56	56.6	
**Tobacco use**				0.173
Current	21 (12.2)	9	42.9	
Former	41 (23.8)	25	61.0	
Never	110 (64.0)	49	44.5	
**Major chronic conditions**				0.003
No	116 (67.4)	47	40.5	
Yes	56 (32.6)	36	64.3	
**Year of contact**				0.772
2022	89 (51.7)	42	47.2	
2023	83 (48.3)	41	49.4	

SAR: secondary attack rate.

The overall SAR was 48.3%. The SAR was higher, and achieved statistical significance in participants 65 years and over (70%), in non-working individuals (61.5%), as well as in subjects who presented some risk factors (56.6%) or chronic conditions (64.3%). No differences in the SAR were found for other variables.

The baseline data concerning preventive measures and SAR are presented in Table 2. Most respondents did not use face mask (59.9%), hand washing more than 4 times per day (57.6%), and ventilated indoor spaces (99.4%) at baseline. In participants without prior COVID-19 infection, the SAR was significantly higher than in patients with prior infection (56.0% vs. 37.5%; *p* = 0.017). However, none of the values in this section achieved statistical significance. Regarding COVID-19 vaccination status, most of the household contacts (93.5%) had two or more doses.

**Table 2:** Baseline variables related to preventive measures and secondary attack rates of household contacts of COVID-19 cases (Spain, May 2022 until July 2023).

	Household contacts	Infections	SAR	*p*-value
	*n* (%)	*n*	%	
**Quarantine**				0.319
No	131 (76.2)	66	50.4	
Yes	41 (23.8)	17	41.5	
**Mask use and type**				0.926
No	103 (59.9)	50	48.5	
Yes	69 (40.1)	33	47.8	
Surgical masks	34 (49.3)	13	38.2	0.284
FFP2 masks	33 (47.8)	19	57.6	
Cloth masks	2 (2.9)	1	50.0	
**Hand washing**				0.196
0 time/day	0	0	0.0	
1–2 times/day	16 (9.3)	9	56.3	
3–4 times/day	57 (33.1)	22	38.6	
>4 times/day	99 (57.6)	52	52.5	
**Hydroalcoholic solution**				0.500
0 time/day	99 (57.6)	46	46.5	
1–2 times/day	21 (12.2)	13	61.9	
3–4 times/day	18 (10.5)	7	38.9	
>4 times/day	34 (19.8)	17	50.0	
**Social distance**				0.070
No	91 (52.9)	51	56.0	
Yes	80 (46.5)	32	40.0	
Unknown	1 (0.6)	0	0.0	
**Ventilation**				0.299
No	1 (0.6)	1	100.0	
Yes	171 (99.4)	82	48.0	
1 time/day	29 (16.9)	14	48.3	0.583
≥2 times/day	142 (82.6)	68	47.9	
**Prior Covid-19 infection**				0.017
No	100 (58.1)	56	56.0	
Yes	72 (41.9)	27	37.5	
**COVID-19 vaccination status**				
Unvaccinated	4 (2.3)	2	50.0	0.090
Vaccinated with 1 dose	7 (4.1)	3	42.9	
Vaccinated with 2 doses	36 (20.9)	13	36.1	
Vaccinated with 3 doses	95 (55.2)	44	46.3	
Vaccinated with 4 doses	30 (17.4)	21	70.0	

SAR: secondary attack rate.

### Changes in knowledge and preventive measures towards COVID-19 over time

A total of one hundred eighteen participants who completed the follow-up survey at three months were included in the present analysis (Figure 1). Our analysis showed that there were no significant differences over time in knowledge (Table 3). Participants were acceptably aware that COVID-19 could be transmitted by an asymptomatic person (85.6% vs. 81.4%; *p* = 0.381), and even if individuals were vaccinated (89.0% vs. 91.5%; *p* = 0.510). Respondents were substantially aware that the virus could spread via respiratory droplets of infected individuals (92.4% vs. 89.8%; *p* = 0.493) in both rounds (at baseline and after the third month). Moreover, participants showed awareness in both rounds that washing their hands (95.8% vs. 94.1%; *p* = 0.553), wearing a face mask, and avoiding crowds in enclosed places (93.2% vs. 90.7%; *p* = 0.473) are effective methods of reducing the virus’ spread.

**Table 3:** Percentage of change in the knowledge about COVID-19 disease and its preventive measures from baseline to post-3 months (Spain, May 2022 until July 2023).

Items and values	Total (*n* = 118)	Household contacts with higher risk of severe COVID-19 (*n* = 66)	Household contacts without risk of severe COVID-19 (*n* = 52)	Household contacts with previous infection (*n* = 50)	Household contacts without previous infection (*n* = 68)
All people who become ill with COVID-19 develop severe cases					
At baseline, %	80.5	74.2	88.5	80.0	80.9
Post-3 months follow up, %	75.4	71.2	80.8	76.0	75.0
% change	−5.1	−3.0	−7.7	−4.0	−5.9
*p*-value	0.346	0.696	0.278	0.629	0.408
Asymptomatic persons diagnosed with COVID-19 can transmit the infection					
At baseline, %	85.6	83.3	88.5	84.0	86.8
Post-3 months follow up, %	81.4	80.3	82.7	86.0	77.9
% change	−4.2	−3.0	−5.8	2.0	−8.9
*p*-value	0.381	0.652	0.402	0.779	0.177
Persons diagnosed with COVID-19 can transmit infection despite vaccination					
At baseline, %	89.0	86.4	92.3	88.0	89.7
Post-3 months follow up, %	91.5	87.9	96.2	90.0	92.6
% change	2.5	1.5	3.9	2.0	2.9
*p*-value	0.510	0.795	0.400	0.749	0.545
The COVID-19 virus is spread by respiratory droplets from infected individuals when coughing/sneezing/talking/laughing/singing					
At baseline, %	92.4	90.9	94.2	94.0	91.2
Post-3 months follow up, %	89.8	87.9	92.3	92.0	88.2
% change	−2.6	−3.0	−1.9	−2.0	−3.0
*p*-value	0.493	0.572	0.696	0.695	0.573
Handwashing is important to reduce the risk of contracting COVID-19					
At baseline, %	95.8	97.0	94.2	98.0	94.1
Post-3 months follow up, %	94.1	95.5	92.3	98.0	91.2
% change	−1.7	−1.5	−1.9	0.0	−2.9
*p*-value	0.553	0.648	0.696	1.000	0.511
To prevent transmission of COVID-19 measures such as wearing face masks and avoiding crowds in enclosed spaces should be maintained					
At baseline, %	93.2	97.0	88.5	94.0	92.6
Post-3 months follow up, %	90.7	95.5	84.6	90.0	91.2
% change	−2.5	−1.5	−3.9	−4.0	−1.4
*p*-value	0.473	0.648	0.566	0.461	0.753

The proportion of respondents who answered correctly that not all individuals with COVID-19 develop severe cases decreased 5.1% (80.5% vs. 75.4%; *p* = 0.346) over time. When participants were categorized into those with higher risk of severe COVID-19 (Table 3), more than 95% of participants in higher-risk categories correctly identified two methods to prevent COVID-19 infection (washing hands and wearing face masks), but no significant differences were found over the two time periods (−1.5%; *p* = 0.648 in both items). In participants with high risk of severe COVID-19 or according to household contacts with or without previous infection, similar changes at three months were observed and no significant changes were found.

### Changes in attitude and preventive measures towards COVID-19 over time

Analysis of attitudes towards COVID-19 were assessed in the 118 participants who completed the three-month follow-up (Table 4). Results showed that participants considered that their inner circle had complied with the non-pharmaceutical preventive measures (86.4% vs. 93.2%; *p* = 0.085) and vaccination recommendations to avoid becoming ill with COVID-19 in both rounds (92.4% vs. 89.0%; *p* = 0.371). Also, while the vast majority of respondents agreed that wearing a face mask correctly is important to prevent COVID-19 in crowded indoor settings, but difference was not statistically significant (95.8% vs. 89.8%; *p* = 0.078). After the third month, the percentage of participants who believed it is better to develop immunity by getting sick with COVID-19 than by being vaccinated increased 5.1% (51.7% vs. 56.8%), but it did not reach statistical significance (*p* = 0.433). Likewise, it was shown that the percentage of those who reported that COVID-19 had a negative influence on their daily lives slightly decreased from 56.8 to 44.1% (*p* = 0.051). The rate of participants’ responses regarding susceptibility to becoming seriously ill if they contracted COVID-19 decreased 4.3% (45.8% vs. 41.5%; *p* = 0.512) over the three months.

**Table 4:** Percentage of change in attitudes about COVID-19 disease and its preventive measures from baseline to post-3 months (Spain, May 2022 until July 2023).

Items and values	Total (*n* = 118)	Household contacts with higher risk of severe COVID-19 (*n* = 66)	Household contacts without risk of severe COVID-19 (*n* = 52)	Household contacts with previous infection (*n* = 50)	Household contacts without previous infection (*n* = 68)
I consider myself susceptible to developing a severe disease if I become ill with COVID-19					
At baseline, %	45.8	24.2	73.1	42.0	48.5
Post-3 months follow up, %	41.5	13.6	76.9	46.0	38.2
% change	−4.3	−10.6	3.8	4.0	−10.3
*p*-value	0.512	0.120	0.651	0.687	0.121
I consider that my inner circle has complied with the preventive measures to avoid becoming ill with COVID-19					
At baseline, %	86.4	86.4	86.5	80.0	91.2
Post-3 months follow up, %	93.2	95.5	90.4	90.0	95.6
% change	6.8	9.1	3.9	10.0	4.4
*p*-value	0.085	0.069	0.539	0.162	0.303
I consider that my inner circle has complied with the vaccination recommendations given by the health authorities					
At baseline, %	92.4	89.4	96.2	90.0	94.1
Post-3 months follow up, %	89.0	86.4	92.3	88.0	89.7
% change	−3.4	−3.0	−3.9	−2.0	−4.4
*p*-value	0.371	0.594	0.400	0.749	0.345
I consider that it is better to develop immunity by getting sick with COVID-19 than by getting vaccinated					
At baseline, %	51.7	51.5	51.9	50.0	52.9
Post-3 months follow up, %	56.8	60.6	51.9	50.0	61.8
% change	5.1	9.1	0.0	0.0	8.9
*p*-value	0.433	0.294	1.000	1.000	0.300
It is convenient for the general population to wear a facemask correctly (covering the nose and mouth) to prevent COVID-19 in crowded closed environments					
At baseline, %	95.8	97.0	94.2	96.0	95.6
Post-3 months follow up, %	89.8	87.9	92.3	90.0	89.7
% change	−6.0	−9.1	−1.9	−6.0	−5.9
*p*-value	0.078	**0.048**	0.696	0.240	0.189
I consider that COVID-19 had a negative influence on my daily life					
At baseline, %	56.8	60.6	51.9	46.0	64.7
Post-3 months follow up, %	44.1	54.5	30.8	28.0	55.9
% change	−12.7	−6.1	−21.1	−18.0	−8.8
*p*-value	0.051	0.481	**0.029**	0.062	0.295

In stratified analysis according to risk to develop severe COVID-19 or taking into account previous infection, similar results were observed. However, a significant decrease of the percentage of respondents without risk of severe COVID-19 who indicated a negative impact in their lives between rounds (51.9% vs. 30.8%; *p* = 0.029).

### Information sources

**Figure 2:**
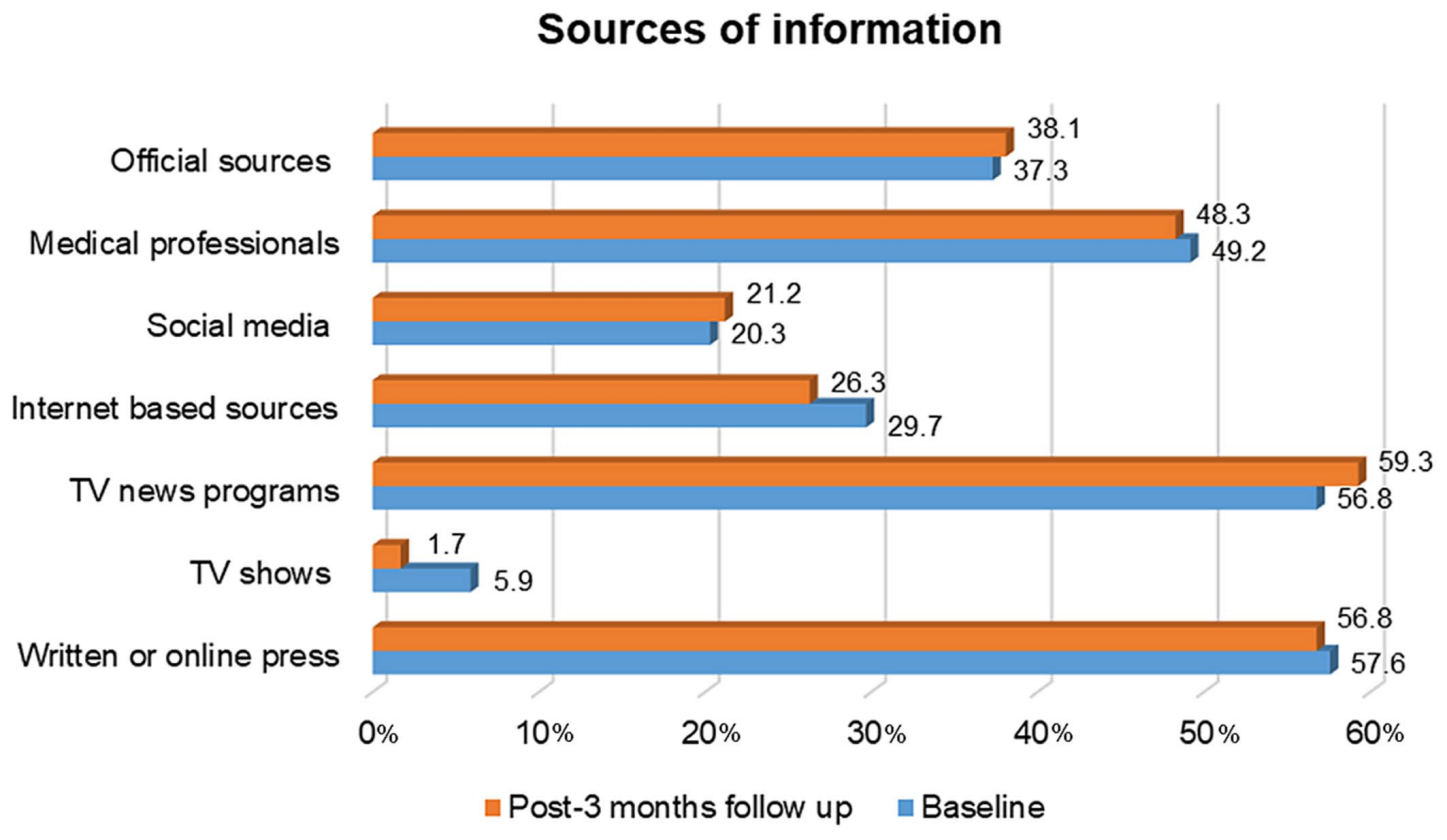
Use of sources of information from baseline to post-3 months (Spain, May 2022 until July 2023).

In addition to knowledge and attitudes concerning COVID-19, we also explored the main sources of information used by participants, as presented in Figure 2. TV programs (56.8% vs. 59.3%) were the most commonly used source of information about COVID-19, followed by the written or online press (57.6% vs. 56.8%), health professionals (49.2% vs. 48.3%), and government or institutional official sources (37.3% vs. 38.1%), at baseline and after the third month, respectively.

## Discussion

The changing nature of COVID-19 has caused preventive measures to be adapted over time. Since 2020, when COVID-19 became a pandemic, governments have implemented several containment measures to reduce the spread of the disease, which included quarantine measures (strict mandatory isolation), the suspension of public transport, and any non-essential economic activity (3–6, 19). Other non-pharmaceutical preventive measures have included the use of face masks, social distancing, large-scale testing, and self-isolation in case of symptoms (3–6, 20, 21). After the number of cases began to decline governments began relaxing COVID-19 restrictions (3, 6). During the study period, in which there was a relaxation of non-pharmaceutical preventive measures, the pooled SAR (48%) was slightly lower than what was observed in a previous study in Spain (58–80%) (22).

The present study explores knowledge and attitudes towards COVID-19 between 2022 and 2023 (from May 2022 to July 2023), in a completely different context from the early days of the pandemic, characterized by a decrease in incidence, no closure constraints, and a relaxation of measures against COVID-19 across two regions of Spain. Our results showed that most household contacts had correct knowledge and attitudes towards COVID-19 during the acute pandemic phase. In line with these results, studies carried out at the beginning of the pandemic in South Asia and in the Middle East (23–25) showed a high prevalence of correct knowledge, attitude and practice (KAP) regarding COVID-19. Given the extensive information campaigns on preventive measures to combat COVID-19, it might be expected that over time, both knowledge about the virus and adherence to preventive measures would improve. However, in the main analysis, we found no significant improvements in knowledge and attitudes over the follow-up at three-months study. The maintenance of knowledge and attitudes over time demonstrates that the population did not show disinterest in the use of non-pharmacological preventive measures and the adherence over the course of the pandemic was consistent to avoided virus spread in the household.

Some studies have reported differences in the way that populations experienced the pandemic, where inequalities in socioeconomic resources seemed to greatly influence access to information, protective equipment, and health care (26–29). A meta-analysis revealed low-income countries had a higher percentage of participants had poor knowledge and weak attitude concerning COVID-19 during the early stages of the pandemic. At the same time the KAP scores were significantly higher in participants in middle age and those with higher education (11). A population that is well-informed in terms of vaccination, mode of transmission and preventive measures is one of the best prevention strategies to fight COVID-19 during this post-acute phase (19).

Our findings suggested that household contacts were aware, both at the baseline and at the three-month follow-up, that SARS-CoV-2 could be transmitted from an infected person even if they are vaccinated or asymptomatic. Most participants also displayed correct knowledge about containment non-pharmaceutical preventive measures such as wearing a face mask and the benefits of vaccination to avoid the spread of COVID-19, which was also constant across the two rounds. Similarly, the vast majority of participants showed correct attitudes about COVID-19.

Our findings are in line with previous COVID-19 research on the Spanish population, which showed high levels of knowledge and good behavior regarding COVID-19 transmission and preventive measures (26). Our results could be influenced by the fact that almost 83% of study participants had secondary school or higher education. Scientific evidence regarding KAP related to COVID-19 suggests that educational status has a positive association with higher percentages of correct knowledge (30). Likewise, other studies have found a different association, where individuals with less education are less likely to adopt preventive measures against COVID-19 (11, 30). Higher rates of knowledge and attitude regarding face mask usage in our population could be attributed to the fact that it was compulsory in Spain to use them in public places such as public transport, health services, and pharmacies from May 2020 to February 2023 (20, 31, 32).

Studies have shown that individuals suffering from chronic illnesses have a greater susceptibility to developing mental health problems, including anxiety and depression, which also increase the risk of hospitalization (33). Taking this into account, adequate KAP within the population is critical to prevent, control, and mitigate infections during COVID-19 outbreaks, especially in demographics with higher-risk conditions (34–36). In our analysis, patients with increased risk of developing severe COVID-19 showed correct knowledge and attitude about COVID-19. Similarly, another study indicated that participants with chronic illnesses had an appropriate level of knowledge and attitude regarding COVID-19, which varied based on several factors (34). Nevertheless, we found that a greater proportion of subjects at higher risk of severe COVID-19 did not consider that COVID-19 could aggravate their health condition in both rounds. A previous study performed on patients with chronic diseases revealed that individuals with more than four comorbidities were significantly less likely to respond that they were more vulnerable than those with one or two chronic illness (36). Outbreaks have been shown to cause a negative impact on mental health, which would increase several physiological symptoms such as stress, anxiety, and depression, which themselves increase vulnerability in populations at high-risk (37). Our data provides a snapshot of the negative impact on daily life (despite the low incidence of cases of COVID-19 and the relaxation of preventive measures), and the limited perception of personal risk in high-risk individuals which would need to be considered to provide effective and relevant messages on protective behaviors for COVID-19 management in these vulnerable populations.

The most popular sources of COVID-19 related information were journalistic sources (TV and written/online press), and less than half of participants (~37%) had trusted information provided by official institutional sources across both rounds. Our results are in line with a study performed on the Swiss population, in which it was found that TV and newspapers were the most popular information sources (38).

Our results could be driven by the fact that 74% of the population was 45 years or older. For instance, Chu et al. (39), revealed that the election of information sources related to COVID-19 was strongly associated with age, where older individuals were more likely to obtain information from multiple sources, especially traditional media (e.g., newspaper and TV), compared to younger subjects. In addition, older individuals were shown to be more likely to perceive the effects of the pandemic as personally relevant, due to greater age-related mortality risks (39). It was pointed out that the reliance on consistent, clear, and trustworthy information from official sources is an essential determinant of correct protective behaviours. Therefore, it would be important to be aware of the information sources and formats that populations habitually use in these contexts to provide strategies to maximise peoples’ trust in official sources. With a greater proportion of the population relying on these sources we will be able to ensure the quality of information, avoid misinformation, and promote better adherence to effective health protective practices.

This study could be of great interest to health authorities and Governments since provide a tool and demonstrated the applicability by which knowledge, attitudes and preventive measures can be monitored, which is essential to evaluate the level of uptake and effectiveness of containment measures implemented to control COVID-19 transmission, and to document changes in knowledge and attitudes over time in the post-acute phase of the pandemic. Additionally, the discrepancies we have identified in how individuals at high-risk of severe COVID-19 perceived the disease’s impact on their health and daily life represents a gap in the research that may warrant further investigation to support effective actions in vulnerable populations.

### Limitations

Our study has some limitations. Although the results of this study are made more robust by the surveying of two distinct population groups in geographically different regions (Catalonia and Navarre). These results could be generalize to the Spanish population since the relaxation of preventive measures against COVID-19 were similar in all regions. However, the results may not be generalizable to other countries, in which similar indications of preventive measures have not been carried out throughout the territory. The low incidence of COVID-19 cases in the post-acute phases of a pandemic may affect the sample size, thereby reducing the certainty of results. The limited sample size did not allow us to perform other sub-analyses. However, each center has performed an in-depth census of household contacts to ensure maximized contact participation, which is reflected in the 75% retention rate of the study. Moreover, this study used a telephone survey method and the information obtained was self-reported, so information biases may have been introduced in the outcome variables, and/ or in the information about vaccination status. Notwithstanding, demographic characteristics, comorbidities, and COVID-19 vaccination status are able to be verified with electronic medical records and vaccination registers, reducing the possibilities of data pollution to near zero. This study evaluate the short-term changes in the knowledge and attitudes at three months when preventive measures were relaxed, and it is still not possible to know what the long-term change is due to the follow-up period of the participants.

## Conclusion

This study showed that household contacts of COVID-19 cases had correct knowledge and positive attitudes during the post-acute phase of the COVID-19 pandemic, in which there was a relaxation of non-pharmaceutical preventive measures. Our findings provide relevant insights regarding to allow researchers, public health professionals, and policymakers for decision-making against the circulation of SARS-CoV-2 or a new health emergency and implement targeted strategies for the general population and vulnerable groups.

## Data availability statement

The raw data supporting the conclusions of this article will be made available by the authors, without undue reservation.

## Ethics statement

The studies involving humans were approved by Bioethics Commission of the University of Barcelona (IRB00003099). The studies were conducted in accordance with the local legislation and institutional requirements. The ethics committee/institutional review board waived the requirement of written informed consent for participation from the participants or the participants' legal guardians/next of kin because all participants provided oral informed consent.

## Author contributions

VB-V: Formal analysis, Investigation, Methodology, Writing – original draft, Writing – review & editing. DT: Conceptualization, Funding acquisition, Investigation, Project administration, Supervision, Writing – review & editing. AE: Data curation, Writing – review & editing. PG: Conceptualization, Data curation, Project administration, Writing – review & editing. MG: Conceptualization, Data curation, Project administration, Writing – review & editing. IP: Conceptualization, Data curation, Writing – review & editing. JC: Conceptualization, Data curation, Writing – review & editing. AD: Conceptualization, Data curation, Writing – review & editing. IM-B: Conceptualization, Data curation, Formal analysis, Funding acquisition, Investigation, Methodology, Project administration, Supervision, Writing – original draft, Writing – review & editing.
